# The neutrophil to lymphocyte ratio (NLR) positively correlates with the presence and severity of metabolic syndrome in obese adults, but not in obese children/adolescents

**DOI:** 10.1186/s12902-023-01369-4

**Published:** 2023-05-26

**Authors:** Alice Marra, Adele Bondesan, Diana Caroli, Graziano Grugni, Alessandro Sartorio

**Affiliations:** 1 Istituto Auxologico Italiano, IRCCS, Experimental Laboratory for Auxo-Endocrinological Research, Piancavallo-Verbania, Italy; 2 Istituto Auxologico Italiano, IRCCS, Division of Auxology, Piancavallo-Verbania, Italy

**Keywords:** Obesity, Metabolic syndrome, Neutrophil to lymphocyte ratio, Adults, Children/adolescents

## Abstract

Metabolic syndrome (MetS) associated with obesity is a pathological condition increasing worldwide. Recent studies have demonstrated that the neutrophil to lymphocyte ratio (NLR) can be successfully used to stage MetS in obese adults. The aim of the study was to evaluate NLR values in 552 children/adolescents (M 219, F 333; 14.8 [12.9–16.3] years) and 231 adults (M 88, F 143; 52.3 [36.4–63.3] years) with morbid obesity, subdivided into subgroups according with the presence or absence of MetS. Adult patients with obesity showed a higher prevalence of MetS compared to the pediatric population (71% vs 26%), associated with a greater number of subjects with 3 and 4–5 altered components for MetS. NLR was higher (*P*-value = 0.041) in adults with MetS compared with those without. NLR values also positively correlated with the severity grade of the syndrome (*P*-value = 0.032). By contrast, in pediatric subjects with obesity with MetS, NLR values were comparable with those recorded in subjects without MetS (*P*-value = 0.861), no correlation being found with MetS severity (*P*-value = 0.441). Our study confirms the importance of NLR as an inflammatory indicator associated with MetS in adult subjects with severe obesity, while it excludes a similar role in children/adolescents.

**Supplementary Information:**

The online version contains supplementary material available at 10.1186/s12902-023-01369-4.

## Introduction

Obesity has reached pandemic proportions worldwide with a rising prevalence of its severe forms both in children and adults [1–3]. Metabolic syndrome (MetS) is a clinical condition frequently associated with obesity, embracing risk factors such as altered glucose metabolism, atherogenic profile, hypertension and abdominal obesity [4, 5]. The occurrence of MetS in patients with obesity has been reported to be significantly correlated with the increase of type 2 diabetes, cardiovascular risks, stroke and non-alcoholic fatty liver disease at all ages [6, 7]. From a pathophysiological point of view, obesity and insulin resistance have been identified as the crucial triggers of MetS development [8, 9].

MetS is considered as a proinflammatory state in which fat excess and visceral adipocytes release chemo-attractants, contributing to macrophages infiltration and release of inflammatory mediators (i.e. cytokines and adipokines), which overall lead to a systemic inflammatory condition [10–12].

Several studies have reported that inflammatory biomarkers (e.g. Interleukin-6, Tumor Necrosis Factor-alpha and C-reactive protein) were significantly increased in adult patients with obesity associated with MetS and that their increment was positively correlated with the severity of MetS, making their evaluation a powerful diagnostic tool, especially during adulthood [13–19].

More recently, the neutrophil to lymphocyte ratio (NLR) has been considered as an indicator of systemic inflammation [20–22] and proposed to detect the presence of MetS and to monitor its severity [23–26].

In this respect, Buyukkaya et al*.* found a significant correlation between the criteria of MetS severity and inflammation on the basis of NLR analysis in a small number of overweight and obese adults [23], while to the best of our knowledge no data is available in obese children and adolescents so far.

Since the prevalence of MetS in obese adults is markedly higher than in obese children/adolescents [27–30] and the alterations of the MetS components are differently represented, thus hypothetically influencing the inflammatory status in a diverse way, the aim of the present study was to investigate the correlations of NLR with the presence and severity of MetS in both adults and children/adolescents with severe obesity.

## Material and methods

### Patients

The study population included 552 obese children and adolescents (219 males and 333 females; median (interquartile range): 14.8 [12.9–16.3] years; median BMI (interquartile range): 36.4 [32.7–40.7]) and 231 adults with obesity (88 males and 143 females, median age (interquartile range): 52.3 [36.4–63.3] years, median BMI (interquartile range): 44.2 [40.4–46.1]), hospitalized at the Division of Auxology and at the Division of Metabolic Diseases, Istituto Auxologico Italiano IRCCS, Piancavallo-Verbania, Italy, respectively, for a 3-week multidisciplinary integrated body weight reduction program (BWRP). In detail, the BWRP consisted of a 3-week in-hospital (i.e. full-time staying in the hospital, including the night) integrated energy-restricted diet in combination with physical rehabilitation (moderate aerobic activity), psychological counseling, and nutritional education.

A Mediterranean diet was prescribed based on the initial basal metabolic rate and physical activity level for each patient. The amount of energy to be given with diet was calculated by subtracting approximately 500 kcal from the measurement of resting energy expenditure. The diet, in terms of macronutrients, contained 21% proteins, 53% carbohydrates, and 26% lipids; the daily estimated water content was 1000 mL, while the estimated salt content was 1560 mg Na^+^, 3600 mg K^+^, and 900 mg Ca^2+^. Extra water intake of at least 2000 mL/day was encouraged. The diet was served in three meals (breakfast at 07.30 AM, lunch at 12.30 PM, and dinner at 07.30 PM). On each day of the BWRP, the patients had dietetics classes consisting of lectures, demonstrations, and group discussions with and without a supervisor.

The physical activity program consisted of five days per week of training, including (i) 1 h dynamic aerobic standing and floor exercise with arms and legs, at moderate intensity and under the guidance of a therapist; and (ii) either 20–30 min cycle ergometer exercise at 60 W, or 3–4 km out-door walking on flat terrain, according to individual capabilities and clinical status.

The subjects also underwent a psychological counseling program consisting of two or three sessions per week of individual and/or group psychotherapy performed by clinical psychologists. Furthermore, lectures on the problems and risks of obesity, motivational speech, examples of healthy foods, foods preparation workshops, and group discussions (with or without a supervisor) took place daily.

In childhood, obesity was defined in presence of a BMI ≥ 97th percentile for gender and chronological age according to the Italian growth charts [31], while in adulthood obesity was defined based on the presence of a BMI > 30 kg/m^2^. Exclusion criteria for patients were: acute or chronic kidney/liver diseases, secondary obesity, acute or chronic infection or inflammatory conditions, autoimmune diseases, malignant diseases, neurodegenerative diseases, hematological and/or oncological disorders.

For each participant, anthropometric and instrumental measurements, metabolic variables were collected.

### Metabolic syndrome definition

According to the IDF criteria for the diagnosis of metabolic syndrome [32, 33], patients with obesity were considered positive for the presence of metabolic syndrome if they had three or more altered factors:

- Adults: (i) abdominal obesity (WC ≥ 102 cm for males; ≥ 88 cm for females), (ii) elevated triglycerides: ≥ 150 mg/dL (1.7 mmol/L) or specific treatment for this lipid abnormality; (iii) reduced HDL-C: < 40 mg/dL (1.0 mmol/L) in males; < 50 mg/dL (1.3 mmol/L) in females or specific treatment for this lipid abnormality; (iv) increased BP: SBP ≥ 130 mmHg or DBP ≥ 85 mmHg and/or treatment of previously diagnosed hypertension; (v) increased fasting plasma glucose (FPG) concentration ≥ 100 mg/dL (5.6 mmol/L) or previously diagnosed type 2 diabetes mellitus.
- Children/Adolescents:
  for children/adolescents aged between 10 and < 16 years: (i) abdominal obesity (WC ≥ 90^th^ percentile), (ii) increased triglycerides: ≥ 150 mg/dL (1.7 mmol/L) or specific treatment for this lipid abnormality; (iii) reduced HDL-C: < 40 mg/dL (1.03 mmol/L); (iv) increased BP: SBP ≥ 130 mmHg or DBP ≥ 85 mmHg and/or treatment of previously diagnosed hypertension; (v) increased fasting plasma glucose (FPG) concentration ≥ 100 mg/dL (5.6 mmol/L) or previously diagnosed type 2 diabetes mellitus.
  for children/adolescents with an age ≥ 16 years: (i) abdominal obesity (WC ≥ 94 cm for males; ≥ 80 cm for females), (ii) increased triglycerides: ≥ 150 mg/dL (1.7 mmol/L) or specific treatment for this lipid abnormality; (iii) reduced HDL-C: < 40 mg/dL (1.03 mmol/L) in males and < 50 mg/dL (< 1.29 mmol/L) in females, or specific treatment for lipid abnormalities; (iv) increased BP: SBP ≥ 130 mmHg or DBP ≥ 85 mmHg and/or treatment of previously diagnosed hypertension; (v) increased fasting plasma glucose (FPG) concentration ≥ 100 mg/dL (5.6 mmol/L) or previously diagnosed type 2 diabetes mellitus.

Patients were subsequently subdivided into three subgroups according to the number of MetS criteria: MetS 1–2 (i.e. the presence of 1–2 MetS criteria = no Mets), MetS 3 (i.e. 3 MetS criteria) and MetS 4–5 (i.e. 4–5 criteria). Patients of subgroup MetS 1–2 were MetS-, while those of subgroups MetS 3 and MetS 4–5 were MetS+ .

### Anthropometric measurements

Physical examination included the determination of height, weight, and waist circumference (WC) by the same trained operators, according to the Anthropometric Standardization Reference Manual [34]. Standing height was determined by a Harpenden Stadiometer (Holtain Limited, Crymych, Dyfed, UK). Body weight was measured to the nearest 0.1 kg using an electronic scale (Ro WU 150, Wunder Sa.bi., Trezzo sull’Adda, Italy). WC was determined in standing position midway between the lowest rib and the top of the iliac crest after gentle expiration, with a non-elastic flexible tape measure [35].

### Laboratory analyses

About 10 mL of blood samples were collected in standard tubes at 8:00 AM after an overnight fast. Blood count and metabolic variables were than determined.

Hematologic parameters were measured using Beckman Coulter instruments. Leukocytes count was performed with the impedance-based method upon erythrocyte (RBC) lysis. Volume, conductivity and scatter properties of leukocytes (VCS Technology) were used to determine leukocytes populations.

Colorimetric enzymatic assays (Roche Diagnostics, Monza, Italy) were used to determine serum HDL-C and triglycerides levels. Serum glucose level was measured by the glucose oxidase enzymatic method (Roche Diagnostics, Monza, Italy). All serum analysis on HDL-C, triglycerides and glucose were performed by using the Roche Cobas 6000 analyzer.

### Blood pressure measurement

Blood pressure (BP) was estimated in subjects in sitting position and relaxed condition using a sphygmomanometer with appropriately sized cuff on the right arm at rest [27]. The procedure was repeated three times and the means of the three values for systolic and diastolic BP were recorded.

### Statistical analysis

Analyses were performed using GraphPad Prism 9 software for Windows (GraphPad Software, San Diego, CA, USA, https://www.graphpad.com/) for data plotting and analysis.

*Shapiro–Wilk* normality test was used to determine the normal distribution and linearity of each variable.

**Fig.1:**
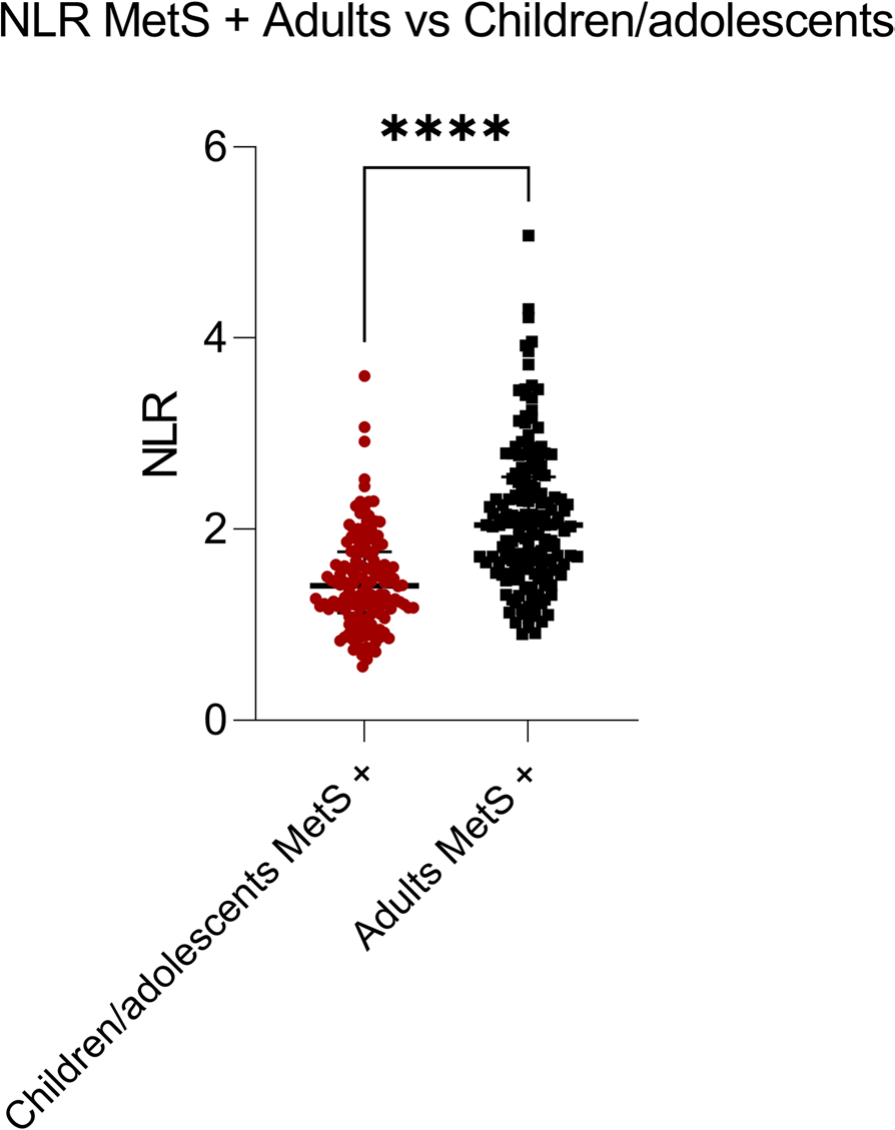
NLR increases in MetS+ obese adults compared with MetS+ obese children/adolescents. Dot plots represent the NLR values in children/adolescents (red) and adults (black) both with metabolic syndrome. The NLR difference in the two populations was calculated using the non-parametric Mann–Whitney U test. **** = *P*-value < 0.0001

**Fig. 2:**
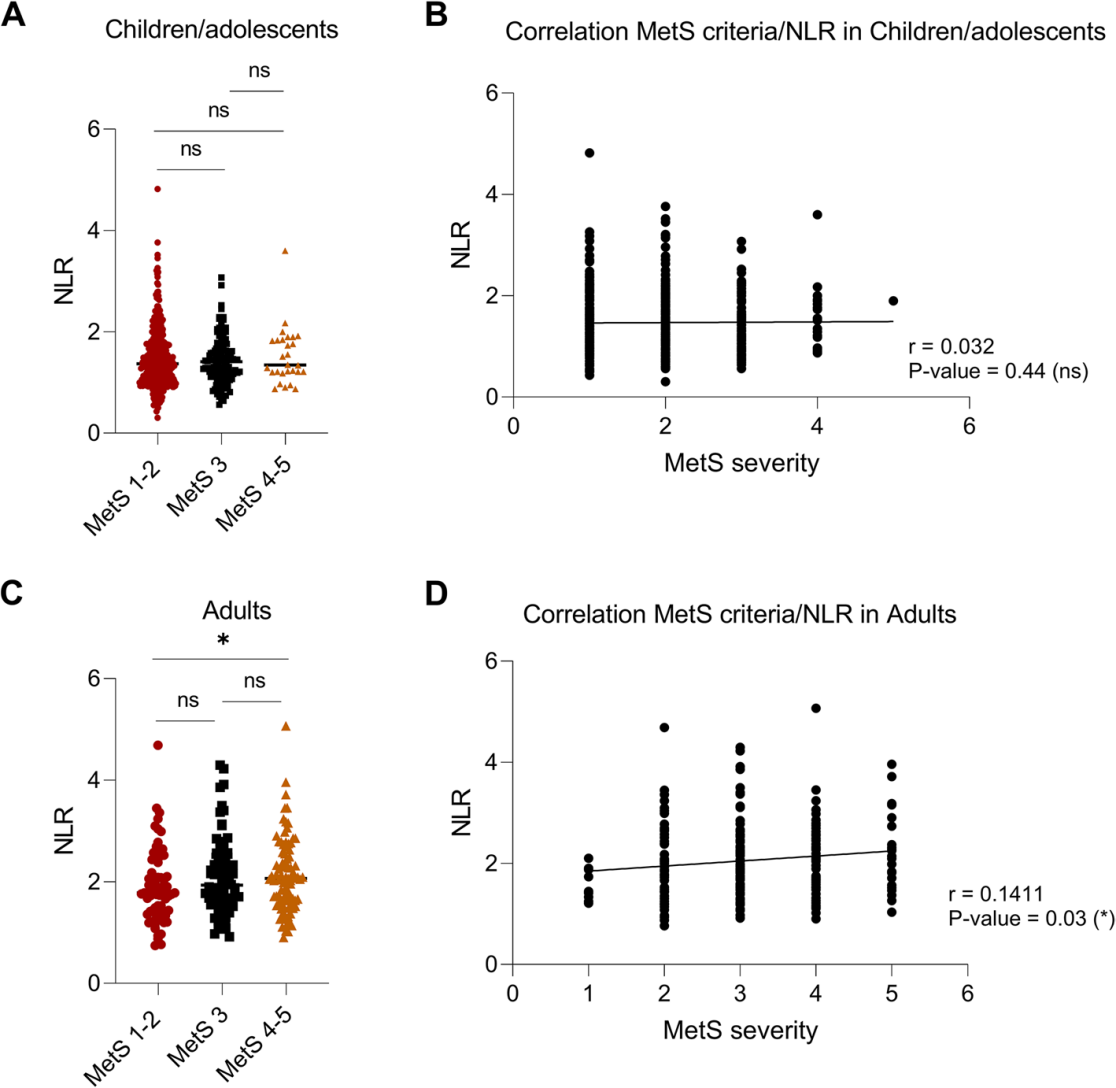
Neutrophils/lymphocytes ratio (NLR) positively correlates with the severity of metabolic syndrome in adult patients. **a**,**c** Dot plots represent the NLR values in children/adolescents and adults according to the severity of metabolic syndrome. The severity of metabolic syndrome is indicated with the amount of IDF criteria: MetS 1–2 (low grade, no metabolic syndrome in red), MetS 3 (moderate grade, in black), MetS 4–5 (high grade in orange). *P*-value is calculated using the non parametric Mann–Whitney U test. * = *P*-value < 0.05; ns (not significant) = *P*-value > 0.05. **b**,**d** Non parametric Spearman rank correlation test between neutrophil-lymphocytes ratio and metabolic syndrome in children/adolescents and adults showed a positively correlation between the variables. Abbreviations: r = correlation coefficient. * = *P*-value < 0.05; ns (not significant) = *P*-value > 0.05

Categorical and continuous variables and in Table 1, Table 2, Table 3, Fig. 1, Fig. 2 and Figure S1 are reported as median (interquartile range) or percentage because of the failure of normalcy.

**Table 1:** Clinical and laboratory parameters of the study populations

**Children/** **adolescents**	**All** **(*N*** **= 552)**	**MetS–** **(*N*** **= 406, 74%)**	**MetS+** **(*N*** **= 146, 26%)**	***p*** **MetS - vs MetS+**	***p*** **M MetS+ vs F MetS+ **
Age, years	14.8 (12.9–16.3)	14.5 (12.4–15.9)	15.8 (14.0–16.9)	< 0.0001	
Sex (N, %)	M 219, 40; F 333, 60	M 151, 69; F 255,77	M 68, 31; F 78, 23	0.523	0.046
BMI, kg/m^2^	36.4 (32.7–40.7)	35.5 (32.2–39.8)	39.3 (35.6–42.6)	< 0.0001	
WC, cm	113.0 (103.0–123.0)	110.0 (101.0–120.0)	122.0 (112.0–132.0)	< 0.0001	
SBP, mm Hg	120.0 (120.0–130.0)	120.0 (110.0–125.0)	130 (130.0–140.0)	< 0.0001	
DBP, mm Hg	80.0 (70.0–80.0)	80.0 (70.0–80.0)	80.0 (80.0–87.5)	< 0.0001	
TG, mmol/L	88.0 (65.0–115.0)	80.5 (62.0–103.3)	117.0 (86.0–158.0)	< 0.0001	
FBG, mmol/L	4.5 (4.3–4.3)	4.5 (4.3–4.3)	4.5 (4.3–4.8)	0.929	
HDL, mg/dL	41.0 (35.0–48.0)	44.0 (39.8–51.0)	35.0 (32.0–38.0)	< 0.0001	
**Adults**	**All** **(*N* = 231)**	**MetS–** **(*N* = 68, 29%)**	**MetS+ ** **(*N* = 163, 71%)**	***p*** **MetS - vs MetS+ **	***p*** **M MetS+ vs F MetS+ **
Age, years	52.3 (36.4–63.3)	44.9 (27.3–62.4)	52.9 (41.5–63.7)	0.027	
Sex (N,%)	M 88, 38; F 143, 62	M 17, 19; F 51, 36	M 71, 81; F 92, 64	0.942	< 0.0001
BMI, kg/m^2^	43.6 (40.5–48.3)	42.7 (40.4–46.1)	44.2 (40.8–48.9)	0.052	
WC, cm	121.0 (114.0–132.0)	115.0 (108.0–126.3)	125.0 (117.0–134.0)	< 0.0001	
SBP, mm Hg	135.0 (120.0–145.0)	125.0 (120.0–140.0)	140.0 (130.0–150.0)	< 0.0001	
DBP, mm Hg	80.0 (80.0–90.0)	80.0 (80.0- 80.0)	80.0 (80.0–90.0)	0.002	
TG, mmol/L	130.0 (102.0–169.0)	104.5 (89.2–129.8)	145.0 (114.0–186.0)	< 0.0001	
FBG, mmol/L	5.4 (4.9–6.1)	5.0 (5.3–4.6)	5.8 (6.8–5.2)	< 0.0001	
HDL, mg/dL	44.0 (38.0–53.0)	51.5 (43.0–59.0)	42.0 (36.0–49.0)	< 0.0001	

Parameters from all obese subjects, without metabolic syndrome and with metabolic syndrome children/adolescents and adults are shown. The total number of the population and the subgroups percentages are indicated. Data is given as median (interquartile range) or %

The gender values represent the % of males and females over the total male and female populations respectively per each condition. WC, SBP, DBP, TG, FBG and HDL are adopted as criteria by IDF to diagnostic metabolic syndrome

*Abbreviations*: *MetS-* Group without metabolic syndrome, *MetS*+ Group with metabolic syndrome, *M* Males, *F* Females, *BMI* Body mass index expressed in kg/m2, *WC* Waist circumference in cm, *SBP* Systolic blood pressure in mm/Hg, *DBP* Diastolic blood pressure in mm Hg, *TG* Triglyceride in mmol/L, *FBG* Fasting blood glucose in mmol/L, *HDL* High-density lipoprotein in mmol/L

*P*-value (*p*) (non parametric Mann–Whitney U test) represents the difference between MetS- vs MetS+ and is considered as significant when < 0.05. Fisher’s exact test was used to assess the effect of gender on the MetS prevalence in children/adolescents and adults respectively (*P*-value = 0.523; *P*-value = 0.942)

**Table 2:** MetS criteria distribution in obese adults and children/adolescents

**MetS criteria**	**Children/adolescents**	**Adults**	***p***
MetS 1–2	N 406; 73.6%	N 68; 29.9%	< 0.0001
MetS 3	N 118; 21.4%	N 72; 31.2%	< 0.0001
MetS 4–5	N 28; 5.0%	N 91; 39.4%	< 0.0001
**MetS criteria males**	**Children/adolescents**	**Adults**	***p***
MetS 1–2	N 151; 68.9%	N 17; 19.3%	< 0.0001
MetS 3	N 57; 26.1%	N 27; 30.7%	< 0.0001
MetS 4–5	N 11; 5.0%	N 44; 50.0%	< 0.0001
**MetS criteria females**	**Children/adolescents**	**Adults**	***p***
MetS 1–2	N 255; 76.6%	N 52; 36.4%	< 0.0001
MetS 3	N 61; 18.3%	N 45; 31.5%	< 0.0001
MetS 4–5	N 17; 5.1%	N 47; 32.9%	< 0.0001

Distribution of metabolic syndrome prevalence grades in the pediatric and adult populations are displayed. Children/adolescents and adults amounts are indicated as total numbers and %. MetS severity grades are differently spread depending on the age. During childhood most of the subjects showed the presence of 1–2 criteria, whereas during adulthood the number of subjects with moderate and high severity of MetS greatly increased (MetS 3, MetS 4–5). *P*-value (p) and differences between the population are calculated with the Fisher’s exact test

**Table 3:** Hematologic parameters of the study populations

**Children/adolescents**	**Obese** **(*N*** **= 552)**	**MetS–** **(*N*** **= 406, 74%)**	**MetS+** **(*N*** **= 146, 26%)**	***p*** **MetS- vs MetS+**
White Blood Cells (10^9^/L)	8.3 (7.1–9.6)	8.2 (7.0–9.6)	8.5 (7.1–9.8)	0.298
Neutrophils count (10^9^/L)	4.2 (3.4–5.2)	4.1 (3.4–5.2)	4.3 (3.4–5.3)	0.190
Lymphocytes count (10^9^/L)	3.0 (2.6–3.6)	3.0 (2.6–3.5)	3.0 (2.6–3.7)	0.379
NLR	1.3 (1.0–1.7)	1.4 (1.1–1.7)	1.41 (1.2–1.7)	0.861
**Adults**	**Obese** **(*N* = 231)**	**MetS–** **(*N* = 68, 29%)**	**MetS+ ** **(*N* = 163, 71%)**	***p*** **MetS- vs MetS+ **
White Blood Cells (10^9^/L)	7.2 (6.1–8.5)	6.8 (5.9–8.4)	7.3 (6.2–8.5)	0.059
Neutrophils count (10^9^/L)	4.1 (3.4–5.0)	3.7 (3.2–4.7)	4.3 (3.5–5.2)	0.023
Lymphocytes count (10^9^/L)	2.1 (1.7–2.5)	2.0 (1.8–2.5)	2.2 (1.7–2.6)	0.911
NLR	1.9 (1.3–2.6)	1.8 (1.4–2.4)	2.0 (1.6–2.5)	0.041

Parameters from all obese subjects, without metabolic syndrome and with metabolic syndrome children/adolescents and adults are shown. The total number of the population and their percentages overall are indicated. Data is given as median (interquartile range) or %

*Abbreviations*: *MetS-* Group without metabolic syndrome, *MetS*+ Group with metabolic syndrome, *NLR* Neutrophils/lymphocytes ratio

*P*-value (*p*) (calculated with the non-parametric Mann–Whitney U test) represents the difference between MetS- vs MetS+ and it is considered as significant when < 0.05

All the parameters were evaluated as continuous variables and compared among all subgroups (obese with/without metabolic syndrome, metabolic syndrome severity grades, children/adults, sex male/female).

The non parametric Mann–Whitney U test was used to compare continuous variables. One-way ANOVA or Kruskal–Wallis tests were used to compare more than two groups. Two-way ANOVA was used to assess how two independent variables (sex and age) affect the NLR values and MetS prevalence. Fisher’s exact test was used to compare contingency tables and categorical variables.

Correlation between the NLR increase and metabolic syndrome severity grade was assessed using non-parametric Spearmen’s rank correlation test.

Raw data of NLR of MetS- and MetS + in adults and children/adolescents is used to draw ROC (receiver operating characteristic) curves and the area under the ROC curves (AUC) was used to assess the accuracy of NLR. The optimal NLR cut-off, the values of area under the curve (AUC), with sensitivity and specificity for the development of MS were calculated.

A level of significance of *P*-value < 0.05 was used for all data analyses.

## Results

The clinical and biochemical data of all subjects morbidly obese, with (MetS+) and without MetS (MetS-) is shown in Table 1.

Both children and adults showed a picture of severe obesity (BMI: 36.4 [32.7–40.7] kg/m^2^, BMI: 43.6 [40.5–48.3] kg/m^2^, respectively). As expected, BMI, triglycerides, systolic and diastolic blood pressure were significantly higher in the MetS+ than in the MetS- group. Waist circumference was significantly higher in the MetS+ group (male children/adolescents < 16 years: 121.5 cm [110.3–128.3], female children/adolescents < 16 years: 113 cm [105.9–125.5]; male children/adolescents ≥ 16 years: 127.0 cm [121.0–138.0], female children/adolescents ≥ 16 years: 119.5 cm [111.5–132.0]; male adults: 133.0 cm [124.0–142.0], female adults: 119.0 cm [113.3–129.8]) than in the MetS- group (male children/adolescents < 16 years: 110.0 cm [100.0–119.9], female children/adolescents < 16 years: 108.0 cm [99.0–116.0]; male children/adolescents ≥ 16 years: 128 cm [116.0–134], female children/adolescents ≥ 16 years: 111.5 cm [104.0–121.0]; male adults: 120.0 cm [113.0–138.0], female adults: 112 cm [106.0–121.0]), whereas serum HDL levels were significantly lower in the MetS+ group (male children/adolescents < 16 years: 34.0 mg/dL [31.0–37.0], female children/adolescents < 16 years: 34.0 mg/dL [34.0–36.0]; male children/adolescents ≥ 16 years: 34.0 mg/dL [30.0–37.0], female children/adolescents ≥ 16 years: 40.0 mg/dL [35.25–42.75]; male adults: 38.0 mg/dL [32.0–43.0], female adults: 46.0 mg/dL [40.0–53.0]) than in the MetS- group (male children/adolescents < 16 years: 43.0 mg/dL [39.2–49.0], female children/adolescents < 16 years: 45.0 mg/dL [40.0–50.5]; male children/adolescents ≥ 16 years: 42.0 mg/dL [39.0–49.0], female children/adolescents ≥ 16 years: 45.5 md/dL [39.0–52.2]; male adults: 43.0 mg/dL [40.5–49], female adults: 54.0 mg/dL [45.0–61.0]), both in adults and in children/adolescents. Fasting blood glucose was higher in MetS+ adults compared with MetS-, while the values were similar in the pediatric group independently from the presence of MetS. Both children/adolescents and adults MetS+ were significantly older than those MetS- (*P*-value < 0.0001 and *P*-value = 0.027 respectively).

According to the age-specific IDF criteria for the definition of MetS, 406 pediatric patients (74%) resulted without MetS and 146 (26%) suffered from MetS. By contrast, a significantly higher prevalence (i.e. diametrically opposite) of subjects MetS+ (71%) was found in adults, only 29% being MetS- (Table 1). Moreover, we found a higher prevalence of MetS+ in the male compared to female population both in children/adolescents and adults (*P*-value = 0.046; *P*-value < 0.0001, Table 1).

Despite the different prevalence of male and female in the pediatric and adult populations, we did not recognize the gender as a discriminating factor for the different distribution of the metabolic syndrome. In the pediatric population, MetS was characterized mainly by the concomitant alteration of WC (*N* = 533, 96.6%), HDL (*N* = 263, 47.6%) and BP (*N* = 232, 42.0%). In adults, the most frequent altered parameters were WC (*N* = 231, 100%), BP (*N* = 179, 77.4%) and HDL (*N* = 125, 54.1%).

In the pediatric population, MetS was characterized mainly by the concomitant alteration of WC (*N* = 533, 96.6%), HDL (*N* = 263, 47.6%) and BP (*N* = 232, 42.0%). In adults, the most frequent altered parameters were WC (*N* = 231, 100%), BP (*N* = 179, 77.4%) and HDL (*N* = 125, 54.1%).

In children/adolescents, MetS 3 criteria and MetS 4-5 criteria were 21.4% and 5.0%, respectively, while in adults the percentages were 31.2% and 39.4%, respectively, reflecting a higher prevalence of MetS in adults than in children with obesity (*P*-value < 0.0001, Table 2).

White blood cells, neutrophils and lymphocytes count and NLR values of obese children/adolescents and adults, with or without MetS, are shown in Table 3. Significantly higher values of NLR were found in obese adults with MetS+ compared with those MetS- (Table 3, *P*-value = 0.041). This result relied upon the increase of the neutrophils count in MetS+ (*P*-value = 0.023), but not upon the lymphocytes count (*P*-value = 0.911). By contrast, no significant differences were found between white blood cells, neutrophils and lymphocytes count and NLR in children/adolescents with or without MetS.

The NLR was markedly increased in the MetS+ adult subjects with obesity compared with the MetS+ pediatric population (Fig. 1, *P*-value < 0.0001). Moreover, the increase of NLR was maintained independently from the sex-ratio when the values were compared between children/adolescents and adults, but no difference was found compared between males and females in the same age group (Figure S1), suggesting that gender is not influential in determining an increase of the NLR. No statistical differences in NLR values between the MetS subgroups were found in obese children/adolescents (Fig. 2A, MetS 1–2 vs 3: *P*-value = 0.949; MetS 1–2 vs 4–5: *P*-value = 0.623; MetS 3 vs 4–5: P-value = 0.565).

NLR values were significantly higher in MetS 4–5 patients compared with those without MetS (MetS 1–2) in adults with obesity (Fig. 2C, *P*-value = 0.042), while no differences were recorded between MetS1-2 group and MetS 3 subjects (Fig. 2C, MetS 3 vs 4–5: *P*-value = 0.707; MetS 1–2 vs 3: *P*-value = 0.136).

A positive correlation between NLR values and the number of MetS criteria was found in adults (Fig. 2D, *P*-value = 0.032), while no correlation was found in children/adolescents (Fig. 2B, *P*-value = 0.441).

**Fig. 3:**
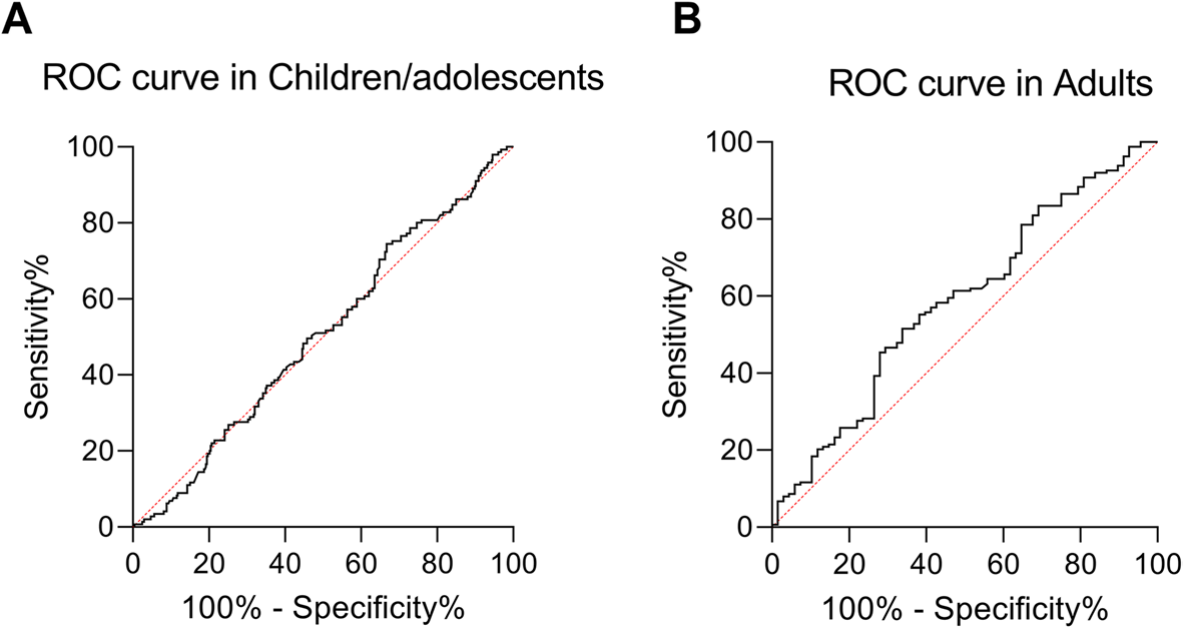
Accuracy of NLR as a marker of metabolic syndrome in children/adolescents and adults. **a** ROC (receiver operating characteristic) curves for NLR in children/adolescents. AUC = 0.502, *P*-value = 0.862. **b** ROC (receiver operating characteristic) curves for NLR in children/adolescents. AUC = 0.5851, *P*-value = 0.041. Abbreviation: ROC, receiver operating characteristic curve; AUC, area under the curve

Finally, the predictive power of NLR in MetS discrimination between age groups was determined. By using the ROC curve analysis, the use of NLR as a continuous variable in obese adults permitted the prediction of MetS with an accuracy of 58.51% (AUC = 0.5851, *P*-value = 0.041), with a sensitivity of 55% and a specificity of 61% at a cut-off of 1.94 (Fig. 3B). On the contrary, MetS in obese children/adolescents was predicted by NLR with an accuracy of 50,04% (AUC = 0.504, *P*-value = 0.861), with a sensitivity of 41.38% and a specificity of 49.52% at a cut-off of 1.48, thus reflecting the lack of difference of NLR between the MetS+ and MetS- subjects (Fig. 3A).

## Discussion

To date,the prevalence of obesity-associated metabolic syndrome (MetS) was reported to be close to 25%—30% in children/adolescents with obesity [27, 29], while in adults the prevalence markedly increased up to 60–65% [28, 30], indicating thus a progressive age-dependent increase of the altered factors determining MetS. Hence, the importance to find new methods for MetS early identification and prevention.

In MetS+ subjects, the adipose tissue constantly releases local and systemic bioactive molecules like adipokines, cytokines and white blood cells detectable by hematic analysis that have been reported to be useful in the diagnosis, follow-up and survey of many systemic inflammatory processes [36, 37].

A recent study has shown that an increased NLR in obese and overweight adults was predictive for the presence of MetS and positively correlated with the MetS grade [23]. However, the analyzed study populations were extremely limited (*N* = 70) and with a relatively low mean BMI (BMI: MetS- = 24.5 ± 4.1; BMI: MetS+  = 29.7 ± 5.9) to infer reliable conclusions. Furthermore, no studies have been performed to evaluate the usefulness of NLR as a precocious biomarker to stage and counter the MetS onset and progression in children/adolescents with obesity so far, which usually displays a shorter duration of obesity and could have different altered determinants for the MetS determination. In this context, the relationship between NLR and BMI in pediatric obesity is still unclear. Mărginean et al. showed that NLR did not differ between obese and normal weight children and adolescents [38]. On the contrary, Aydin et al. showed that NLR was significantly increased in obese adolescents compared with health controls [39]. With this background, the aim of our investigation was to evaluate NLR in a large group of severely obese adults (*N* = 231) and children/adolescents (*N* = 552), subdivided in three subgroups (MetS 1–2, MetS3 and MetS 4–5) on the basis of the altered components determining the MetS presence and severity.

In the present study, we demonstrated that NLR acts as a biomarker for MetS diagnosis in adult subjects with morbid obesity, being significantly higher in MetS+ patients with obesity compared with MetS- (*P*-value = 0.041). By contrast, we showed that NLR values were comparable in MetS+ children/adolescents compared with their MetS- counterpart indicating that the altered parameters concurring to determine MetS in this pediatric population (waist circumference, low HDL high blood pressure) were unable to affect NLR.

NLR values were significantly higher in adults than in children/adolescents with MetS (*P*-value < 0.0001), the ratio being influenced by the increase of the neutrophils count in MetS+ , while the lymphocytes count was comparable in the two subgroups. The statistical significance between the two groups was maintained even after correction of NLR by BMI (*P*-value < 0.001), suggesting that the biomarker difference between the two populations does not depend on adiposity per se (BMI), but likely on the inflammatory state which become chronic in adults compared to children/adolescents.

Although it is well documented that obesity causes a chronic low-grade systemic inflammation, which is stronger when associated with MetS [40], the results of the present study seem to indicate that this condition manifests itself with less impact in childhood, since NLR did not differentiate the subgroup with or without MetS. A different degree of inflammation occurred in obese adults, when the prevalence of MetS was markedly higher compared to the childhood (71%, 26% respectively) and the duration of obesity was likely longer.

The different behavior of NLR values in children/adolescents vs adults with or without MetS might be temptatively explained by the different altered parameters determining MetS, by the simple age advancement (i.e. duration of the disease and greater release of inflammatory citokynes [41–43]), by the progressive worsening of MetS prevalence or by a combination of the previous factors.

According to our previous observations [27, 28], MetS prevalence in the present study population was higher in males than females, both in children/adolescents and adults (children/adolescents: M 31%, F 23%, *P*-value = 0.046; adults: M 81%, F 64%, *P*-value < 0.001), thus excluding a gender-related influence in the age-related different behavior.

Despite the important evidence of our work and the large study population recruited in a single third level center for severe obesity, there are also several limitations that need to be taken into consideration.

First, our data in children/adolescents and adults are not longitudinal and, thus, do not allow to identify the chronological order of the MetS onset and development and their impact on the patients’ health. Furthermore, we have not collected information regarding the start and duration of MetS treatment. Second, although most potentially confounding factors were controlled, we cannot exclude the possibility that MetS could be affected by other lifestyle variables which are related to the concentrations of NLR.

Lastly, although our data demonstrated that NLR positively correlates with the presence and severity of MetS in obese adults, the ROC analysis showed a limited accuracy of NLR as a MetS clinical predictive biomarker. Other biochemical markers will have to be studied for this purpose.

In conclusion, the lower NLR values found in obese children/adolescents than in adults, suggest a lower degree of systemic inflammation, resulting independently from the severity of the disease. This finding might represent an additional proof for a more easily reversible clinical picture in childhood after an early body weight reduction program. Further additional studies are requested in order to better understand the age-dependent different relationships between NLR and MetS found in adults and children/adolescents.

## Supplementary Information

**Additional file 1:** **Supplementary Figure 1**. NLR comparison according to gender in Children/adolescents and Adults.

## Data Availability

The datasets used and/or analysed during the current study are available from the corresponding author on reasonable request on Zenodo repository (http://www.zenodo.org).

## Abbreviations

MetS: Metabolic syndrome
BMI: Body mass index
NLR: Neutrophil to lymphocyte ratio
HDL: High-density lipoprotein
FPG: Fasting plasma glucose
BP: Blood pressure
SBP: Systolic blood pressure
DBP: Diastolic blood pressure
WC: Waist circumference
TG: Triglycerides
ROC: Receiver operating characteristic curve
AUC: Area under the curve
