# Supplementary material for: The neutrophil to lymphocyte ratio (NLR) positively correlates with the presence and severity of metabolic syndrome in obese adults, but not in obese children/adolescents
Source: BMC Endocr Disord. 2023 May 26;23:121. doi: 10.1186/s12902-023-01369-4 (PMC10224327; doi:10.1186/s12902-023-01369-4)
Supplement: Supplementary file 1 — Additional file 1: Supplementary Figure 1. NLR comparison according to gender in Children/adolescents and Adults. [file 12902_2023_1369_MOESM1_ESM.pdf]

Supplementary Figure 1. NLR comparison according to gender in Children/adolescents and Adults.

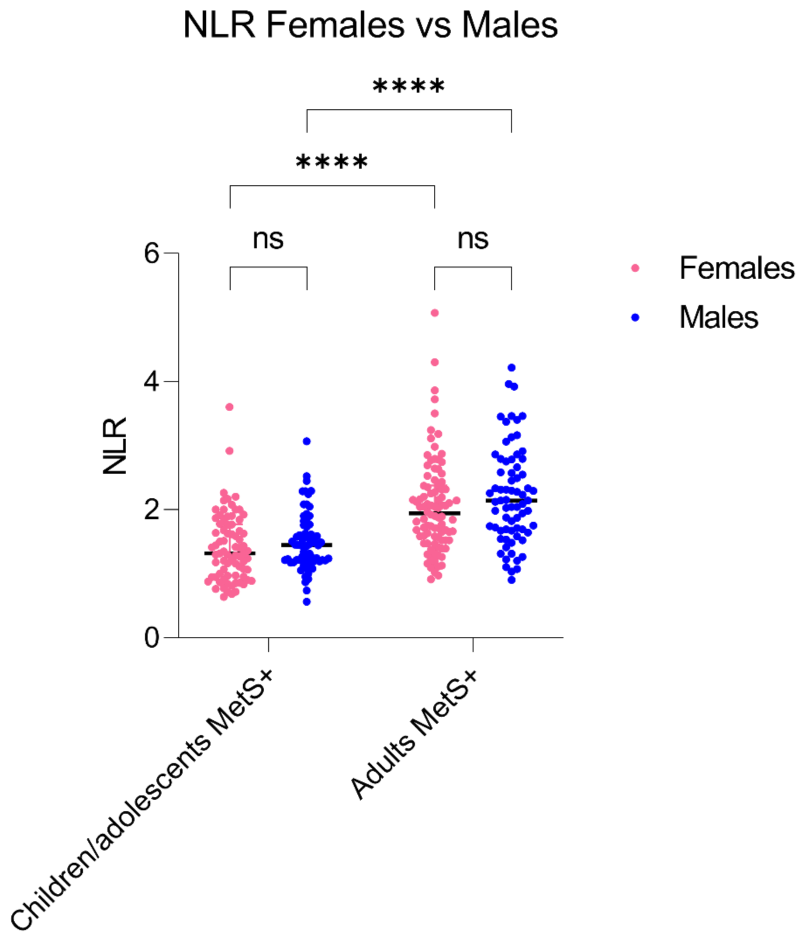

Dot plots represent the NLR values in children/adolescents and adults both with metabolic syndrome according to the gender. The values are represented as median and the NLR difference between the populations was calculated using the non parametric Mann-Whitney U test. **** = P-value < 0.0001.
